# 

**Published:** 1896-02-01

**Authors:** 


### CONTENTS

**The Dead Weight of the Sick** . . . . . 291

**The Colliery Disaster** . . . . . 292

**Clinical Recollections.** By Sir B. W. Richardson, M.D. . 293

**Modern Sociology.**—The Demoralising Tendency of Reformatory and Industrial Schools . . . . . 294

**Medical Progress and Hospital Clinics—**

Hæmorrhoids: Causes and Symptoms (*continued*) . . . 297

Physical Treatment in Heart Disease . . . . 298

Progress in Medicine.—Infective Diseases (*continued*) . 299

Progress in Surgery.—Cerebral Surgery . . . . 300

New Appliances and Things Medical . . . . 301

**Annotations.**—On Sudden Death—From Butter to Brains—Trades Unionism in Medicine . . . . . 295

**Notes and News** . . . . . 296

**Anthropology at the Universities** . . . . . 302

**The Institutional Workshop—**

Hospital Construction.—Trimmer's Cottage Hospital, Farnham . . . . . 303

Practical Departments . . . . . 304

Construction Notes . . . . . 304

**Scraps and Gleanings** . . . . . 304

**The Student of Medicine** . . . . . 305

### EXTRA SUPPLEMENT.—THE NURSING MIRROR.

**News from the Nursing World.**—Our Courteous Queen—The Nurses' Co operation—Scrubbers as Assistant Nurses—Working Hours at Lockerbie—Nursing Soldiers' Families—District Nurses at Paisley—Coventry Nursing Institution—Sunderland Nursing Institute—Well Cared-for—On the Labrador Coast—Les Enfants Assistés—Short Items . . . cxlvii

**Lectures on Nursing. VII.**—Ventilation . . . . cxlix

**Entertainments at the Hospitals** . . . . cl

**The Royal British Nurses' Association** . . . cli

**Nursing in Florence.** III.—Spedale Degli Innocenti (Foundling Hospital) . . . . . cl

**The "Oriolet" Hospital** . . . . . cliv

**Wants and Workers** . . . . . cliv

**Everybody's Opinion** . . . . . clv

**Notes and Queries** . . . . . clv

**For Reading to the Sick** . . . . . clvi

**Where to Go** . . . . . clvi

**Appointments** . . . . . clvi

